# Talking body: the effect of body and voice anthropomorphism on perception of social agents

**DOI:** 10.3389/frobt.2024.1456613

**Published:** 2024-10-09

**Authors:** Kashyap Haresamudram, Ilaria Torre, Magnus Behling, Christoph Wagner, Stefan Larsson

**Affiliations:** ^1^ Department of Technology and Society, Lund University, Lund, Sweden; ^2^ Department of Computer Science and Engineering, Chalmers University of Technology, Gothenburg, Sweden; ^3^ Department of Economics, Lund University, Lund, Sweden

**Keywords:** human-agent interaction, social robot, voice assistant, human factors, trust, anthropomorphism, CASA paradigm

## Abstract

**Introduction:**

In human-agent interaction, trust is often measured using human-trust constructs such as competence, benevolence, and integrity, however, it is unclear whether technology-trust constructs such as functionality, helpfulness, and reliability are more suitable. There is also evidence that perception of “humanness” measured through anthropomorphism varies based on the characteristics of the agent, but dimensions of anthropomorphism are not highlighted in empirical studies.

**Methods:**

In order to study how different embodiments and qualities of speech of agents influence type of trust and dimensions of anthropomorphism in perception of the agent, we conducted an experiment using two agent “bodies”, a speaker and robot, employing four levels of “humanness of voice”, and measured perception of the agent using human-trust, technology-trust, and Godspeed series questionnaires.

**Results:**

We found that the agents elicit both human and technology conceptions of trust with no significant difference, that differences in body and voice of an agent have no significant impact on trust, even though body and voice are both independently significant in anthropomorphism perception.

**Discussion:**

Interestingly, the results indicate that voice may be a stronger characteristic in influencing the perception of agents (not relating to trust) than physical appearance or body. We discuss the implications of our findings for research on human-agent interaction and highlight future research areas.

## 1 Introduction

Trust is often considered fundamental to human social activity (Fukuyama, 1995). It is an implicit, everyday reality (Rotter, 1980) that influences social activity on an individual as well as systemic level (Luhmann, 2018; Paxton, 2007). Trust plays a pivotal role in forming and maintaining human relationships (Fehr, 1988; Rempel et al., 2001). Research shows that trust is also crucial to human interaction with non-human artefacts (Gram, 2024; Vance et al., 2008; Benbasat and Wang, 2005). It is no surprise then that trust has become a central topic of research in human-technology relationships (Saariluoma et al., 2019).

The multidisciplinary field of Human-Computer Interaction (HCI) has produced significant research on trust in relation to various forms of technology (Karat et al., 2007; Carolus et al., 2019; Warner-Søderholm et al., 2018). Recent technical advancements in artificial intelligence (AI) and automation technologies (AT) have highlighted the need for a more human-centric, interaction-based approach to technology development (Shneiderman, 2020; Wang et al., 2020), and consequently a re-examination of trust concepts in HCI (Madhavan and Wiegmann, 2007). This is further made visible in the more recent field of Human-Agent/Robot Interaction (HAI/HRI)^1^ (Lazányi and Hajdu, 2017), which is broadly related to, and influenced by, HCI (Heuer and Stein, 2020; Hancock et al., 2011) and especially relevant to interaction with social agents.

Social agents are unique due to their primary purpose of fulfilling a social role in interaction with humans (Henschel et al., 2021). Seeing as these artifacts are designed to be social actors, discourse regarding an influential HCI theory, the ‘Computers Are Social Actors Paradigm’ (CASA paradigm), has re-emerged in HAI research (Gambino et al., 2020). The notion that computers are perceived as social actors by humans has been a long-stay in HCI (Lombard and Xu, 2021). The CASA paradigm proposes that humans have a tendency to apply the same social rules and heuristics when interacting with computers as they do when interacting with humans (Nass et al., 1994; Nass and Moon, 2000). Based on the assumption that humans prefer and find it intuitive to socially interact with non-human agents that look and behave like humans, social agent/robot design has often involved intentionally building human-like characteristics into the physical appearance and/or behaviours of agents (Fink, 2012). This approach is substantiated by observed human psychological phenomenon such as anthropomorphism–the tendency to attribute human qualities to non-human entities (Złotowski et al., 2015), highlighting a bias towards human-centric interaction with the environment. And research has shown that anthropomorphism has a positive effect on perceptions of competence and trustworthiness in autonomous technologies (Waytz et al., 2014; De Visser et al., 2016; Gong, 2008).

However, generally, the multidimensional nature of anthropomorphism (arising from different sensory experiences in humans), though sometimes theoretically discussed (Fink, 2012), is not often empirically highlighted in HAI research. Instead, anthropomorphism is often treated (and measured) as a unidimensional phenomenon (Li and Suh, 2021). Visuo-centric anthropomorphism is the most extensively studied, starting from computers (Kim and Sundar, 2012), to digital personas (Nowak and Rauh, 2008), and physical robots (Fink, 2012). The body of an agent has thus received considerable attention. The advent of conversational agents has stoked research interest in voice-based anthropomorphism (Seaborn et al., 2021), for example, with regard to gender (Seaborn and Pennefather, 2022). But as agents become more complex and multimodal, it becomes important to explicitly highlight the multidimensionality of the consequent anthropomorphism^2^, identify and study the relationship between various dimensions, and understand how they influence human perception in human-agent interaction. It is as-yet unclear whether there is a more nuanced relationship between the various dimensions of anthropomorphism and trust perception.

Aiming to contribute to the growing knowledge on multidimensional anthropomorphism and trust in human-agent interaction, we conduct an experiment with the purpose of studying (1) the type of trust exhibited by humans in social agents, and (2) how body and voice modalities of an agent interact with one another to influence perception of the agent, particularly trust and anthropomorphism. We manipulate the modality of the agent, body (voice assistant speaker vs. humanoid robot) and voice (four levels of humanness of speech) of the agent (see Section 3.3), and measure the perception of trust, anthropomorphism, and other social characteristics (animacy, likeability, perceived intelligence and perceived safety) of the agent. Through this, we study whether users report human-trust or technology-trust when watching an interaction video of a social agent with varying combinations of body and voice, whether the two modalities (body and voice) differently influence the perception of trust, and whether they differently influence the perception of anthropomorphism (highlighting its different dimensions).

In this paper, we briefly overview the background for the study in Section 2, elaborate on the methods and materials used for the experiment in Section 3, present the results of the experiment in Section 4, and lastly, discuss the broader implications of the results, limitations of the study, and future research opportunities in Section 5.

## 2 Background

In order to contextualise the study and its results, we first review some of the theoretical concepts that our hypotheses (see Section 3.2) are founded on. We draw from research on the CASA paradigm, trust, anthropomorphism and embodiment, particularly in relation to HCI and HAI, as a theoretical framework for the study design (Section 3.3). Additionally, we briefly elaborate on related experimental research on anthropomorphism in HAI.

### 2.1 CASA paradigm

In their seminal work *The Media Equation: How People Treat Computers, Television, and New Media Like Real People and Places*, Reeves and Nass (1996) argue that humans do not automatically distinguish between real life and mediated representations, prompting *mindless* (instinctive or lacking in deliberation) and *social* responses when interacting with mediated representations that mimic human social characteristics (Reeves and Nass, 1996). The CASA paradigm emerged from this discourse, more narrowly focused on human interaction with technologies that are perceived to exhibit social cues and some level of agency (Nass et al., 1994; Nass and Moon, 2000).

Several studies have found evidence to support CASA. Nass, who co-proposed the original theory, was part of several studies that showed that humans reacted socially to computers when they exhibited social cues; people perceived computers more positively when the computer was labelled as a teammate compared to when it was not (Nass et al., 1994), they preferred computers that flattered them (Fogg and Nass, 1997), and they applied human gender stereotypes onto computers that presented a gendered voice (Nass et al., 1997). Apart from computers, CASA has also found support across various technologies such as smartphones (Carolus et al., 2019), chatbots (Adam et al., 2021), voice assistants (Schneider and Hagmann, 2022), robots (Kim et al., 2013), and autonomous vehicles (Waytz et al., 2014). Various studies have found that a wide variety of social cues, even subtle ones, can result in humans reacting socially to various forms of technology. As a result, CASA has had a considerable impact on HCI research.

At the same time, there have been several critiques of CASA over the years, ranging from critiques of its applicability a quarter of a century after it was proposed, given the drastically evolving relationship between humans and computers (Lang et al., 2013), inconsistent reproducibility of CASA in interaction with computers (Schaumburg, 2001), to calls for more nuanced understanding regarding where and how such a phenomenon might occur during interaction (Tzeng, 2006). Alternatively, it has been argued that the CASA paradigm may uniquely apply to emerging technologies (mindless interaction) and diminish as humans gain a better understanding of the technologies they interact with (mindful interaction) (Heyselaar, 2023).

The CASA paradigm has nevertheless endured in the HAI context for two reasons: (1) The two boundary conditions required by the framework–social cues and perceived agency (Gambino et al., 2020), are largely satisfied by social agents (Erel et al., 2019; Brincker, 2016); (2) Social agents are more likely than other technologies to mirror the human-human interaction (HHI) paradigm due to the very nature of their design (Oguz et al., 2018; Raković et al., 2024). As a result, it could be argued that the CASA paradigm is more robust in the HAI context than it is in HCI. Theoretical frameworks to expand and update CASA in light of AI and robotics have already been proposed (Gambino et al., 2020; Lombard and Xu, 2021). Some have even argued that social (collaborative) robots *need* to be social as well as emotional actors in interaction to successfully serve their intended purpose (Fischer, 2019). On the flip side, early empirical research shows that, similar to HCI, evidence for CASA in HAI is far from conclusive, with counter-intuitive findings that even social agents may not always be perceived as social actors by users (Fischer, 2011). Whether social agents are perceived as social actors has implications for trust in them (Fischer, 2011), and consequently for their design and development.

### 2.2 Trust

Trust is a broad, complex, multifaceted concept, that is difficult to define and measure. It is a fundamental human phenomenon that encapsulates internal (Gill et al., 2005), interpersonal (Lahno, 2004), and societal (Falcone and Castelfranchi, 2001) aspects, which interact with and influence one another (Robbins, 2016). Trusting behaviour is an individual characteristic (Sutherland et al., 2020), as well as a situational and interpersonal variable (Ellonen et al., 2008). As a result, there is no real scientific consensus on the mechanisms of trust, with several theoretical perspectives from various disciplines attempting to explain various aspects of it (Simon, 2020). Beyond humans, trust has been used to understand human experience of abstract, non-human artefacts, such as objects (Gram, 2024), corporations (Turnbull, 2003), and systems (Jalava, 2003).

Recently, ‘trust in technology’ has been an important and growing area of research. While it has been argued that the concept of trust in technology is meaningless as technology itself is not a concrete thing upon which to place trust (Pitt, 2010), it is generally understood that trust in technology as a whole is markedly different from (interpersonal) trust in humans (Nickel et al., 2010; Li et al., 2012). However, with regard to specific technologies as opposed to the general concept of technology, there is less consensus about the nature of trust, particularly in technologies possessing human-like qualities (Gambino et al., 2020). Lankton et al. (2015) note that some research on trust in technology has used human-trust concepts such as ability, benevolence, and integrity, that are used to measure interpersonal trust in humans, while others have used technology-trust concepts such as reliability, functionality, and helpfulness, often used to measure trust in technological artefacts (Lankton et al., 2015). Choosing a trust concept is determined by whether a technology is perceived to possess human-like qualities. The CASA paradigm as a consequence has had considerable impact on trust research in humanoid machines, as it lends itself to the use of human-trust concepts. HRI research has produced some evidence for human interpersonal trust concepts in robots (Christoforakos et al., 2021).

Schaumburg (2001) notes two strands of theory on interaction with computers that can be contradictory to one another, the social and the cognitive (Schaumburg, 2001) – arguably equating to CASA and non-CASA respectively. While the social perspective emphasises social rules within interaction design to improve approachablility and understandability, the cognitive perspective assumes that the primary goal of interaction is to accomplish a given task, emphasising efficiency, control and predictability instead (Schaumburg, 2001). In a similar vein, Seeger et al. (2017) highlight a duality in HCI trust research, ones that align with CASA, and ones that disagree with the premise that computers are social actors like humans, arguing instead that humans have distinctly different expectations of, and thus type of trust in, computers compared to humans (Seeger et al., 2017). Non-CASA research in HCI has given rise to a distinctly separate construct for technology-trust from that of human-trust (that CASA research generally employs) (Gebru et al., 2022). While the two types of trust concepts are analogous to one another, they emphasise distinctly different explanations for the formation of trust. These explanations can have a significant impact on the design of technology, including autonomous agents. While it is likely that social agents elicit both human-trust and technology-trust, as observed with some other social technologies (Lankton and McKnight, 2011), it remains an open question how differences in ‘humanness’ of agents are perceived, and whether it influences the type of trust. In our study, we explore this question by employing the ability/competence, benevolence and integrity (ABI or CBI) framework to measure human-trust, and its analogous functionality, helpfulness, and reliability (FHR) conceptualisation to measure technology-trust (see Section 3.4.1).

### 2.3 Anthropomorphism and embodiment

Anthropomorphism is the phenomenon where humans attribute human qualities to non-human entities (Złotowski et al., 2015). While the phenomenon is widely observed, the extent of its prevalence is not clear–given that there is considerable variability between humans as well as contexts (Waytz et al., 2010), and the triggers for anthropomorphism are not exhaustively defined, as it often involves various sensory, affective, and perceptual characteristics in varying combinations (Li and Suh, 2021) – making it a complex, multidimensional phenomenon. Irrespective, anthropomorphism is a central concept in HAI. On one hand, humanoid social agents today are capable of speaking, expressing, and moving (to some extent) in human-like ways (Leite et al., 2013), on the other, agents remain narrowly specialised in their capabilities. Research on how anthropomorphism in humanoid agents is perceived, and what this perception means for HAI, is a developing frontier. As autonomous systems become ubiquitous in society, this research is crucial for safe and purposeful implementation of these systems.

There are several simultaneous and overlapping phenomena at play in human visual perception that are biased towards anthropomorphism. Firstly, we seem to be hardwired for pattern recognition, resulting in the perception of real world objects within abstract shapes. For example, we often observe various familiar shapes and patterns in our environment, such as seeing objects and animals in cloud formations. This phenomenon is known as *Pareidolia* (Liu et al., 2014). Secondly, *Facial Pareidolia* seems to be a strong effect, where we interpret faces within objects in our surroundings with minimal cues. For example, cars are often described as having faces, and emoticons (the precursors to emojis) are interpreted as faces. Thirdly, own-species bias seems to also play an important role, where we are more likely to perceive something to be a human face as opposed to an animal, and we humanise even animal faces and expressions (Scott and Fava, 2013).

Anthropomorphism has been leveraged as a tool in social agent interaction design (Fink, 2012). Theoretically, it is argued that humanoid agents prime certain interaction affordances that are intuitive to humans by aligning with familiar HHI mechanisms (Fink, 2012). This is especially highlighted in the design of the head/face of social robots, often with eyes and mouth similar to that of humans, providing an interaction focal point to emulate HHI (Fink, 2012). While a significant portion of research on anthropomorphism in HAI pertains to the physical representation or body of an agent (referred to as body anthropomorphism in this paper), it is generally agreed upon that behaviours such as gestures and speech also trigger anthropomorphism (Salem et al., 2011). However, studies on anthropomorphism generally view it as a singular phenomenon and do not often address its various dimensions and the interplay between them. Part of the reason perhaps is that anthropomorphism has come to be closely associated with the concept of embodiment (Roesler et al., 2021), which is often viewed (at least in HAI) as a simplistic binary–embodied vs. disembodied (Dautenhahn et al., 2002). In this conception, the focus of perception (of embodiment) is external rather than internal, i.e., it is from the point of view of what is visible (visuo-centric) rather than felt or perceived (Mondada, 2016). Such a conception leads to over-emphasising certain aspects of anthropomorphism while downplaying others. For example, it is still debated whether voice alone can lead to anthropomorphism in light of inconsistent empirical findings, and more research is needed in this direction (Li and Suh, 2021).

Correia et al. question this by asking, “ [c]an we consider that voice itself is an anthropomorphic feature and that it is enough for humans to create an embodied mental model of an agent?” (Correia et al., 2020). While the question needs further enquiry, there is a strong theoretical argument to be made in favour. Complex speech has thus far only been observed in humans (Simonyan et al., 2016), and language is a complex phenomenon that has played a crucial role in the evolution of human cognition, knowledge structures and communication (De Stefani and De Marco, 2019). As a result, speech and language are strong markers for human cognition and intelligence (Perlovsky, 2009) as well as social interaction (Mondada, 2016). Not only that, language is inherently embodied relative to human experience (Dove, 2023). It is also highly metaphorical, with references embedded in human social ontology (Lakoff and Johnson, 1980). And is loaded with cultural and contextual knowledge (Borghi et al., 2019). As a consequence, it can be argued that when an agent is able to speak^3^, it carries and conveys all these intrinsic human qualities of speech and language, eliciting anthropomorphic perception (referred to as voice anthropomorphism in this paper) independent of the physical presentation of an agent. Such a conception would encourage categorising voice assistants (and even chatbots without digital representations) as *perceptively embodied*. If body and voice both enable embodiment, and anthropomorphism, it is then important to compare and contrast voice anthropomorphism with body anthropomorphism, and study how they individually contribute to as well as interact with one another in the perception of social agents. In our study, we employ a robot and a speaker, that both produce four different levels of humanness of voice (see Section 3.3.1), in order to study this.

### 2.4 Relevant studies

Our study builds on top of existing experimental research findings on anthropomorphism and trust in HAI. In a meta-analysis on the effectiveness of anthropomorphism in HRI, covering 78 studies with approximately 6,000 participants overall, Roesler et al. (2021) found that anthropomorphism had a medium (size) positive effect on humans interacting with robots, including a medium (size) positive effect on perception of trust (Roesler et al., 2021). Similarly, Blut et al. (2021) conducted a meta analysis on anthropomorphism in service provision through various agent types (robots, chatbots and other AI), covering 108 independent samples with approximately 11,000 participants overall, and found that anthropomorphism has a positive effect on trust (as a relational mediator) (Blut et al., 2021). Particularly in voice assistants, Hsu and Lee (2023) showed that human-like linguistic and behavioural traits increased trust (Hsu and Lee, 2023). Introducing more nuance to trusting behaviour, Chen and Park (2021) found that anthropomorphism did not directly induce trust in intelligent personal assistants, rather it increased social attraction or task attraction, which in turn reinforced cognitive or affective trust (Chen and Park, 2021). In terms of voice, Torre et al. (2020) showed that a ‘smiling voice’ increased and sustained trust, despite evidence against trustworthiness, in vocally expressive agents (Torre et al., 2020). Foehr and Germelmann (2020) identify perceived personality of the technology’s voice interface as one of four paths (and the only anthropomorphism-based path) to trust in smart voice-interaction technology (Foehr and Germelmann, 2020). In terms of agent embodiment, in a scenario-based study using service chatbots in banking, Kim and Im (2023) showed that users perceived highly intelligent but disembodied agents as more human-like compared to highly intelligent agents with poorly designed appearances (Kim and Im, 2023). Lawson-Guidigbe et al. (2020) found that different visual embodiment of virtual agents resulted in varying levels of trust in an autonomous car context, with the mechanical-human model being rated as the most trustworthy, followed by the human model (Lawson-Guidigbe et al., 2020). Schneiders et al. (2021) showed that for embodied personal assistnats, anthropomorphism did not affected the perception of overall goodness, but had an impact on the perception of Perceived Intelligence, Likeability, and the Pragmatic Qualities of the device (Schneiders et al., 2021). Similarly, Luo et al. (2023) showed that embodiment influenced perception of anthropomorphism in voice assistants, and that physical robots with voice elicited higher anthropomorphism than voice-only assistants (Luo et al., 2023). These studies have all established a relationship between anthropomorphism (and embodiment), trust, and perception. In our study, we contribute to this body of work by further exploring agent body and voice as two dimensions of anthropomorphism, their relationship to one another, and their impact on trust and perception of agents.

## 3 Methods and materials

As elaborated, trust, anthropomorphism and the CASA paradigm are seemingly connected in HAI. The argument being that anthropomorphism results in the perception of human attributes in non-human artefacts, and the CASA paradigm states that humans use human-social interaction mechanisms (trust among them) when interacting with agents perceived to exhibit human social cues, thus resulting in human-trust in humanoid agents. In this paper, we contribute to the understanding of this relationship by conducting an experiment to answer the following research questions: (1) Do humans exhibit different types of trust (human-trust vs. technology-trust) in social agents? (2) Do different modalities of agent (body and voice) have an impact on the type of trust exhibited by humans? and (3) What is the impact of body and voice modalities on (dimensions of) anthropomorphism perception, and other social characteristics?

### 3.1 Conditions

The dependent variables in this setup are the participants’ level of human-trust, technology-trust, anthropomorphism, animacy, likeability, perceived intelligence and perceived safety of the agent. The independent variables are two categories of the physical *body* of the agent, (1) *speaker body–SB* (2) *humanoid robot body–RB*, and four categories of humanness of the *voice* ^4^ ^5^, (1) *text-to-speech voice - TSV*, (2) *AI generated voice–AIV*, (3) *modified human voice - MHV*, and (4) *authentic human voice–AHV*, resulting in a *2x4 factorial design*. Human-trust (HT) and technology-trust (TT) are both repeated measures as well as independent measures variables (since both are measured using one scale across conditions and the differences are analysed within each condition as well as across–see Section 3.4.1), whereas all other dependent variables are independent measures only. The interaction effects between the independent variables is of interest. The eight conditions comprising the study detailed in this paper are denoted as follows.

• RB-AHV: Robot Body with Authentic Human Voice

• RB-MHV: Robot Body with Modified Human Voice

• RB-AIV: Robot Body with AI Generated Voice

• RB-TSV: Robot Body with Text-to-Speech Voice

• SB-AHV: Speaker Body with Authentic Human Voice

• SB-MHV: Speaker Body with Modified Human Voice

• SB-AIV: Speaker Body with AI Generated Voice

• SB-TSV: Speaker Body with Text-to-Speech Voice

### 3.2 Hypotheses

From this study design, we formulate the following 22 hypotheses to guide our data analysis. To reiterate, the independent variables are agent body (SB and RB), and agent voice (AHV, MHV, AIV and TSV)^6^, giving rise to the eight aforementioned conditions. The hypotheses are formulated for each of the seven dependent variables, human-trust, technology-trust, anthropomorphism, animacy, likeability, perceived intelligence, and perceived safety.

#### 3.2.1 Trust hypotheses

• **H_1:** There is no difference between human-trust and technology-trust scores in all conditions

• **H_2a:** There is no difference between human-trust scores for agent body

• **H_2b:** There is no difference between human-trust scores for agent voice

• **H_2c:** The effect of agent body on human-trust score does not depend on agent voice (and *vice versa*)

• **H_3a:** There is no significant difference between technology-trust scores for agent body

• **H_3b:** There is no significant difference between technology-trust scores for agent voice

• **H_3c:** The effect of agent body on technology-trust score does not depend on agent voice (and *vice versa*)

#### 3.2.2 Anthropomorphism hypotheses

• **H_4a:** There is no significant difference between anthropomorphism scores for agent body

• **H_4b:** There is no significant difference between anthropomorphism scores for agent voice

• **H_4c:** The effect of agent body on anthropomorphism score does not depend on agent voice (and *vice versa*)

#### 3.2.3 Animacy hypotheses

• **H_5a:** There is no significant difference between animacy scores for agent body

• **H_5b:** There is no significant difference between animacy scores for agent voice

• **H_5c:** The effect of agent body on animacy score does not depend on agent voice (and *vice versa*)

#### 3.2.4 Likeability hypotheses

• **H_6a:** There is no significant difference between likeability scores for agent body

• **H_6b:** There is no significant difference between likeability scores for agent voice

• **H_6c:** The effect of agent body on likeability score does not depend on agent voice (and *vice versa*)

#### 3.2.5 Perceived intelligence hypotheses

• **H_7a:** There is no significant difference between perceived intelligence scores for agent body

• **H_7b:** There is no significant difference between perceived intelligence scores for agent voice

• **H_7c:** The effect of agent body on perceived intelligence score does not depend on agent voice (and *vice versa*)

#### 3.2.6 Perceived safety hypotheses

• **H_8a:** There is no significant difference between perceived safety scores for agent body

• **H_8b:** There is no significant difference between perceived safety scores for agent voice

• **H_8c:** The effect of agent body on perceived safety score does not depend on agent voice (and *vice versa*)

### 3.3 Experimental setup

The study was conducted online using the participant recruitment platform Prolific (www.prolific.com). The video-based experiment design, and data collection, was done using Sunet Survey (www.sunet.se). Video-based and scenario-based study design (Xu et al., 2015) is relatively commonplace in HAI as it offers the ability to collect larger samples of data, mitigate technical issues with agents, and simulate technically challenging interactions. Studies have shown that video-based HAI study results can be comparable to live HAI study results (Woods et al., 2006; Honig and Oron-Gilad, 2020). Recruited participants were first asked to provide consent for participation, some demographic information was collected, followed by a vignette, and lastly, a video of a human-agent interaction. After watching the video, they were asked to answer questionnaires (see Sections 3.4.1-2). The experiment took 20 min, on average, to complete. The participants were compensated (above average) according to Prolific’s calculations, in-line with acceptable hourly wages in Sweden (although participant recruitment was global–see Section 3.4.3).

#### 3.3.1 Vignette

The study employed a ‘vignette’ (see Supplementary Appendix 1) to incite the appropriate setting and elicit consistent behaviour from the participants for data collection. Vignettes are short descriptions of situations that allow participants to situate the study in a real-world context, enabling more contextually relevant responses in survey studies (Atzmüller and Steiner, 2010). In this study, the participants were informed that the agent was a travel assistant called ‘Otto’, that was capable of making all reservations pertaining to a trip, and that the video is of a user interacting with the agent to plan a vacation. In the videos, a user (only user voice is heard on the video–see Section 3.3.1) interacts with Otto to plan a vacation. The vignette depicts a successful interaction (see Supplementary Appendix 1). This would control for performance-related trust effects. The scales were adapted in order to allow individuals to place themselves in the user’s position while answering the questions (see Supplementary Appendix 2). Through this, the study examined the participants’ perception of trust, anthropomorphism, and other measured dependent variables, of the agent. Since the agent is a speaker in some conditions and robot in others, the agent was named ‘Otto’ in order to maintain consistency when referring to it across conditions. The travel assistant context was chosen in order to present a plausible real world application of an agent that was not too invasive in terms of privacy (such as banking or insurance).

#### 3.3.2 Videos

The videos were made from separately recorded audio and video files that were edited together to simulate an interaction. Apple iMovie was used to combine and edit the audio and video files. A single video file was created using AHV audio. The video was then duplicated for all other conditions, ensuring consistency. The videos were 3 min in length.

##### 3.3.2.1 Video recordings

**FIGURE 1:**
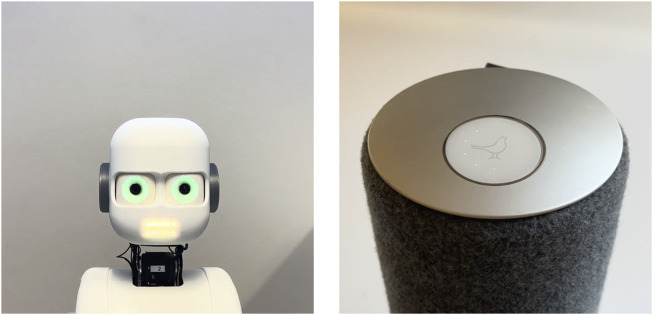
Screenshots of robot and speaker from videos.

A generic speaker and a humanoid robot were used to simulate interaction in the videos (Figure 1). Videos of the speaker and the robot were recorded with no audio using a smartphone. The speaker is equipped with a touch panel on top, containing a glowing logo and a ring of dotted lights surrounding the logo. The logo and the lights were manipulated to convey two modes, listening and speaking. To convey listening the lights circled around the logo, and to convey speaking, the lights pulsated along with the logo. The robot used for the experiment was the Epi robot platform developed at LUCS, Lund University (Johansson et al., 2020). The eyes and mouth of the humanoid robot are equipped with LED lights, which were manipulated to convey listening and speaking. The lights in the eyes were made to glow brighter when speaking and dimmer when listening, and the lights in the mouth were made to pulsate when speaking and switching off while listening.

##### 3.3.2.2 Audio recordings

A script was written according to the vignette (see Supplementary Appendix 1) which was used to record all four voices. All conditions used a male voice and were recorded in English. A female human voice was recorded separately as the human user interacting with the agent. We decided to have a female user in order to more distinctly separate the human voice from the agent voice–especially in the AHV condition. Gender of the voice was kept constant as a control, but gender effects are not within the scope of the study. The built-in text-to-speech generator on Apple MacOS 12 Monterey was used for the TSV condition. NaturalReader’s (www.naturalreaders.com) ‘AI text-to-speech generator’ was used for the AIV condition^7^. While they are both text-to-speech (TTS) voices, the former is a lot less advanced than the latter resulting in markedly different voice ‘humanness’. Existing consumer TTS applications were chosen in an effort to improve external validity. Lastly, a real human voice was recorded for the AHV condition. The AHV audio was modified using several effects on audacity to mimic a robotic voice for the MHV condition. Since the AHV (also consequently MHV) and user voice recordings had some background noise caught by the microphone, the clean audio files from the TTS applications were played out-loud and re-recorded through the same microphone to replicate similar background noise. This ensured consistency across conditions, as well as aided the vignette by making the final videos mimic real-world recordings.

To test for significance of perceived difference between the four chosen voices, 216 participants were asked to listen to a 15 s audio clip of each voice and rate them on a five-point Likert scale with ‘artificial-lifelike’ end-nodes (a single item taken from the Godspeed anthropomorphism scale). A repeated measures Friedman test was performed to find that there was a statistically significant difference in perception of the voices, $\left(\chi^{2}=491.159,p<.001,df=3\right)$ , with the highest mean ranks for AHV, followed by AIV, TSV, and MHV (AHV=3.72,AIV=3.03,TSV=1.73,MHV=1.51) ^8^. Post hoc analysis using paired-sample sign tests were conducted with a Bonferroni correction applied, resulting in a significance level set at (p=.0083) . There was a significant difference between all six pairs (p<.001) , leading us to conclude that the voices reflect distinctly different levels of humanness as intended for the study.

The female user voice was edited together with the AHV, MHV, AIV and TSV audio (individually) to mimic a human-agent conversation, resulting in the complete human-agent interaction audio for the four conditions, which were used as the audio in the final videos.

### 3.4 Measurement

Existing scales were used to measure human-trust, technology-trust, anthropomorphism, animacy, likeability, perceived intelligence, and perceived safety. The trust scales were adapted to fit the vignette and the online study design. The Godspeed scales did not require adaptation.

#### 3.4.1 Trust perception

Trust was measured using an adapted version of the human-trust and technology-trust scales developed by Lankton et al. (2015) based on the competence, benevolence, and integrity (CBI) framework for human-trust, and functionality, helpfulness, and reliability (FHR) concepts for technology-trust (Lankton et al., 2015). Although the sub-scales for perceived usefulness, enjoyment, trusting intention, and continuance intention were also measured, they are not reported in the analysis. Only the three respective sub-scales for CBI and FHR are reported and interpreted in the analysis. Other studies have previously employed some of the trust items on the scale (Law et al., 2022). The technology humanness sub-scale was not used since anthropomorphism is measured separately. Regarding the distinction between human-trust and technology-trust, Lankton et al. (2015) claim that, “literature is not clear whether contexts exist in which using one set of trust constructs is more or less appropriate than using the other. It could be that each type’s influence on outcome variables depends on users’ perceptions of a technology’s human-like characteristics. If these perceptions matter to trust, the choice of whether to use a human-like or a system-like trust concept (and its measures) may make a crucial empirical difference” (Lankton et al., 2015). This makes the scale especially suited to this study, particularly since the scale was build to support a view that technology humanness is a continuum from technology-like to human-like, which is a particularly relevant conceptualisation in this study. A precursor to this scale was employed to study Facebook trusting belief to show that people distinguish the technology-like trust characteristics of Facebook from its human-like trust characteristics, and that all six trusting belief types are discriminant from one another (Lankton and McKnight, 2011).

#### 3.4.2 Anthropomorphism perception

Anthropomorphism was measured using the Godspeed Series (Bartneck et al., 2009). It has been used widely and consistently in HRI research. The series consists of five questionnaires measuring anthropomorphism, animacy, likeability, perceived intelligence, and perceived safety. While the anthropomorphism scale is of primary interest, all the other scales measure some quality of sociability of the agent, so they are also relevant–we refer to them collectively as ‘social characteristics’. The original scales did not require to be adapted.

#### 3.4.3 Participants

The data was collected one condition at a time over a period of 2 weeks. The following conditions were applied using the inbuilt filter on prolific.

• Participants must be native-level speakers of English

• Participants must have access to a device with audio

• Participants must not have taken part in other conditions of the experiment

• Participants must be evenly split by gender

A total of 800 participants were recruited, 100 per condition. Invalid data points were removed during pre-processing. The final sample size, split between the conditions, was.

• RB-AHV: 99; SB-AHV: 99

• RB-MHV: 95; SB-MHV: 100

• RB-AIV: 97; SB-AIV: 98

• RB-TSV: 100; SB-TSV: 97

The highest education level for just under half of the participants was a bachelors degree at 47%, followed by high school degree at 21%, and masters degree at 16%. A majority of the participants reported being proficient at using technology, with 57% identifying as moderately proficient, followed by 30% as extremely proficient with technology. A majority of the participants were also familiar with virtual assistants. About 51% reported using a virtual assistant (such as Google assistant or Amazon Alexa) sometimes, 35% reported using them often, and 11% reported never using them.

### 3.5 Analysis

The data was measured using Likert-scales (7-point scales for Human-trust and technology-trust, and five-point scales for anthropomorphism, animacy, likeability, perceived intelligence and perceived safety). Likert-scales present a couple of different options in terms of analysis. Parametric statistics, such as t-tests and ANOVA tests (which are of relevance to this study), are only viable for interval-scale variables. However, it has been argued that “parametric statistics can be used with Likert data, with small sample sizes, with unequal variances, and with non-normal distributions, with no fear of ‘coming to the wrong conclusion’. These findings are consistent with empirical literature dating back nearly 80 years” (Norman, 2010). This assumption of interval scaling of rating scales has become established in research practice (Döring and Bortz, 2016; Mummendey and Grau, 2014). However, some statisticians insist the ordinal nature of Likert data, and favour the use of non-parametric tests such as Friedman tests and Kruskal–Wallis tests. In our analysis, we perform a combination of the two, favouring parametric tests where possible, due to their higher power, and using non-parametric alternatives where necessary (see Section 3.5.1).

Before we calculated the single scores for each of the sub-scale items to use for the analysis, we checked whether the sub-scale items were sufficiently consistent to indicate a reliable measure of the construct using Cronbach’s Alpha. As can be seen in Table 1, the Cronbach’s Alpha scores for all sub-scales are greater than 0.7, which is deemed satisfactory, the only exception being perceived safety, which is just under for some of the conditions. However, Cronbach’s Alpha is sensitive to the number of items on a scale, and the Godspeed Perceived Safety scale is comprised of only three items, which explains the lower scores. Given that the scale has not been adapted, is widely used, and the Cronbach’s Alpha scores are not significantly below 0.7 (with most above 0.6), we consider it acceptable.

**TABLE 1:** Cronbach’s Alpha scores.

	RB	RB	RB	RB	SB	SB	SB	SB
	AHV	MHV	AIV	TSV	AHV	MHV	AIV	TSV
Competence	0.921	0.935	0.970	0.955	0.920	0.930	0.927	0.933
Benevolance	0.769	0.755	0.864	0.912	0.824	0.840	0.809	0.848
Integrity	0.869	0.919	0.927	0.924	0.873	0.855	0.861	0.916
Human-Trust	0.903	0.885	0.944	0.948	0.888	0.910	0.902	0.933
Functionality	0.874	0.951	0.958	0.937	0.853	0.909	0.911	0.891
Helpfulness	0.794	0.796	0.715	0.874	0.817	0.866	0.831	0.830
Reliability	0.883	0.876	0.912	0.922	0.890	0.903	0.857	0.896
Technology-Trust	0.901	0.901	0.920	0.934	0.897	0.895	0.887	0.917
Anthropomorphism	0.839	0.771	0.837	0.845	0.798	0.853	0.747	0.796
Animacy	0.828	0.719	0.863	0.778	0.848	0.842	0.826	0.818
Likeability	0.901	0.883	0.917	0.891	0.945	0.912	0.888	0.859
P. Intelligence	0.849	0.878	0.889	0.915	0.894	0.862	0.811	0.849
P. Safety	0.667	0.634	0.617	0.719	0.648	0.689	0.593	0.738

Having established the internal reliability of the scales, the parametric tests are performed on the mean value of the Likert-scale items, whereas the non-parametric tests are performed on the median values. This is in keeping with how interval and ordinal data are treated in parametric and non-parametric statistics respectively. For the parametric tests on human-trust and technology-trust, the mean value of individual sub-scale items for competence-benevolence-integrity (CBI), and functionality-helpfulness-reliability (FHR) were calculated, the mean of these individual-item-means then represents each of these constructs. The mean value of the construct-level means for CBI represents human-trust score and the mean value of the construct-level means for FHR represents technology-trust score. Similarly, the mean value of individual sub-scale items for anthropomorphism, animacy, likeability, perceived intelligence and perceived safety were calculated, the mean of these individual-item-means then represents the respective scores. The median-based scores were calculated in a similar manner for the non-parametric tests.

#### 3.5.1 Tests and preconditions

Paired-sample t-tests are used to compare two matched pairs of dependent variables, such as Human-trust and Technology-trust within each condition in this study. The preconditions (1) interval or ratio-scaled dependent variables, (2) two related-group independent variable, and (3) absence of outliers, were satisfied. However, (4) normally distributed differences of paired, dependent variables, was not satisfied. As a result, we cannot perform t-tests. Instead, we perform their non-parametric equivalent, the Wilcoxon signed-rank test. The preconditions for this test, (1) Ordinal or continuous-scaled dependent variables, (2) two categorical, related-group, independent variables, and (3) symmetrically distributed differences of paired, dependent variables, are satisfied by the data.

Two-way ANOVA tests allow analysing both main and interaction effects of two or more independent variables on a dependent variable, which is necessary in this study to understand the relation between independent variables body and voice, and their individual as well as combined effect on the dependent variables. The study design ensures the preconditions (1) independence of measurements, (2) nominal-scaled independent variables, and as per the argument above, (3) interval-scaled dependent variables. The precondition, (4) absence of outliers, was deemed inapplicable though the data contains extreme values with more than 1.5 standard deviations above or below the median, as all valid data-points in this exploratory study are to be considered relevant. However, both (5) Gaussian distributed residuals of dependent variables–or normality, and (6) homogeneity of variances of dependent variables–or homoscedasticity, were not satisfied in all the conditions. This is a common occurrence with real-world data. Consequently, variance-stabilizing transformations according to Box-Cox (Box and Cox, 1964) were applied (see Table 2). ANOVA tests are generally robust against moderate deviations to normality assumption, as simulation studies using a variety of non-normal distributions have shown that the false positive rate is not affected much by this violation of the assumption (Glass et al., 1972; Harwell et al., 1992; Lix et al., 1996). Similarly, homogeneity of variance (homoscedasticity) could not be assumed in all conditions. Where this precondition is not fulfilled, variance-stabilizing transformations (Box and Cox, 1964) were applied. In case of persisting heteroscedasticity, which was found to be common in our data (see Table 3), a robust two-way ANOVA (generalized pivotal quantity or gPQ based generalized test) was employed (Ananda et al., 2023) to mitigate the issue. In cases where all preconditions were satisfied, we only report the two-way ANOVA results, in cases where non-normality persisted post-transformation, we mention the number of conditions that do not fulfil the precondition, and in cases where homoscedasticity is not achieved post-transformation, we report the robust two-way ANOVA results. Games-Howell *post hoc* tests were performed and reported where applicable. The preconditions for this test are the same as preconditions (1), (2), and (3) for the two-way ANOVA, which are satisfied.

**TABLE 2:** Shapiro-Wilk normality test - pre and post data transformation.

	Data	RB	RB	RB	RB	SB	SB	SB	SB
	Transf	AHV	MHV	AIV	TSV	AHV	MHV	AIV	TSV
Human-Trust	Pre	.010	.040	.007	<.001	.007	.013	.005	.016
	Post	.672	.888	.554	.566	.523	.733	.366	.925
Technology-Trust	Pre	.002	.109	.004	.001	.120	.067	.084	.076
	Post	.388	.109	.895	.404	.120	.067	.084	.076
Anthropo-morphism	Pre	.035	<.001	.065	<.001	.118	<.001	.583	.005
	Post	.012	<.001	.203	.002	.012	<.001	.221	.032
Animacy	Pre	.089	.061	.337	.105	.175	.042	.092	.442
	Post	.096	.061	.337	.105	.175	.042	.092	.442
Likeability	Pre	<.001	.146	.003	.002	<.001	.008	.001	.026
	Post	.005	.022	.001	.007	<.001	.010	.017	.289
Perceived Intelligence	Pre	<.001	<.001	<.001	<.001	<.001	<.001	<.001	<.001
	Post	<.001	.044	.003	<.001	<.001	.006	<.001	.005
Perceived Safety	Pre	.006	.093	.007	<.001	<.001	.049	.004	.045
	Post	.039	.083	.005	.013	.007	.022	.018	.063

**TABLE 3:** Levene’s Homogeniety Test (on transformed data) - MHV-included and MHV-excluded.

Condition	MHV-included		MHV-excluded	
	Levene Statistic	Sig	Levene Statistic	Sig
Human-trust	1.852	.075	2.359	0.39
Technology-trust	1.466	.176	1.995	.078
Anthropomorphism	1.912	.065	1.996	.082
Animacy	1.350	.224	.827	.531
Likeability	2.391	.020	2.313	.043
Perceived Intelligence	1.659	.116	2.295	.044
Perceived Safety	1.217	.290	1.086	.369

Given the persistence of non-normality and heteroscedasticity in our data despite transformation (see Tables 2, 3) in some cases, there is a risk that the significance of the ANOVA result is over-estimated. Though robust ANOVA reduces the risk, in order to account for this and supplement the ANOVA results, non-parametric tests on the non-transformed data were also performed. We only report these additional tests where the ANOVA preconditions are not satisfied, and the ANOVA indicates significant results. We employ the Mann-Whitney U test with body as the independent variable, and Kruskal–Wallis test with voice as an independent variable, along with Holm-Bonferroni adjusted pairwise Dunn test. We determine that no further tests are required for interaction effects between body and voice, as elaborated in the results. The preconditions for the Mann-Whitney U test, (1) independence of measurements (2) ordinal or continuous-scaled dependent variable, and (3) one categorical, independent group, independent variable, are all satisfied by the data. The preconditions for Kruskal–Wallis test, (1) independence of measurements (2) ordinal or continuous-scaled dependent variable, and (3) two or more categorical, independent group, independent variables, are all satisfied by the data. The fourth and final precondition (4) similar shape distributions of groups, is not satisfied. However this assumption only needs to be satisfied when testing for a difference between medians, but does not need to be satisfied for testing dominance between distributions.

The data was analysed using a combination of Microsoft Excel, IBM SPSS, and RStudio. We produced data visualisations only where a statistically significant result was observed due to a limitation on tables and figures. Visualisations were created using R packages ‘ggpubr’ (Alboukadel, 2023) and ‘ggstatsplot’ (Patil, 2021).

#### 3.5.2 Problems and mitigation

It was discovered during analysis that the MHV audio produced confounding results. As was found in the individual rating of the four voice-snippets, the MHV audio ranked last when compared to the other three voices, potentially indicating a design flaw. This reflected in the results for the full study (see Section 4) as well. Upon further inspection of the free-text remarks made by the participants at the end of the survey, it was found that 15 and 17 participants for RB-MHV and SB-MHV, respectively, made unfavourable remarks concerning the MHV voice (no such remarks were made regarding any of the other voices). The remarks broadly fell into three categories, (1) the voice was unclear, (2) the voice was weird, and (3) the voice was unpleasant. The overly edited nature of the voice, in order to make AHV-audio sound robotic, has seemingly caused the voice to have explicitly negative reactions, which is not intended. This would cause some difficulty in interpreting the results. In order to mitigate this problem and draw meaningful inferences from the results, we analysed the data both with and without the two MHV conditions, RB-MHV and SB-MHV. We present results from analysing both the MHV-included dataset as well as the MHV-excluded dataset. Exclusion of the MHV conditions does not significantly alter the study design, or its ability to produce meaningful insights towards the research questions. In light of this, the MHV-excluded results are to be considered the primary results of the study and its research questions. While we discuss how the MHV-included results could be interpreted in light of other existing research, we do this in order to explain any differences between the MHV-included and MHV-excluded results. We intend to present the results from both the original data (MHV-included) along with the data adjusted to address design issues (MHV-excluded), to ensure research transparency.

## 4 Results

For the parametric tests, we report the results of two analyses, using *MHV-included dataset* and *MHV-excluded dataset*, for each independent variable. Post-hoc tests for ANOVA are performed only on MHV-included dataset, as the pairwise tests can be interpreted both with and without the MHV conditions. However, we only use MHV-excluded results for hypothesis testing.

For the non-parametric tests, we report MHV-included results for human-trust vs. technology-trust, as the results can be interpreted both with and without the MHV conditions. We only report MHV-excluded results of the non-parametric alternative test to an ANOVA^9^, as it is not of relevance for the MHV-included dataset. Non-parametric test results are only reported where statistical significance is found in the two-way ANOVA results. Any contradictions between the tests are also reported.

### 4.1 Human-trust vs. technology-trust

A pairwise, Wilcoxon signed-rank test was performed to compare human-trust and technology-trust pairs in all eight conditions (RB-AHV, RB-MHV, RB-AIV, RB-TSV, SB-AHV, SB-MHV, SB-AIV, and SB-TSV). The results shown in Table 4 indicate no statistically significant difference in ranks between human-trust and technology-trust pairs in any of the groups. As a result, the null hypothesis H_1 – there is no significant difference between human-trust and technology-trust scores for RB-AHV, RB-MHV, RB-AIV, RB-TSV, SB-AHV, SB-MHV, SB-AIV, and SB-TSV, is accepted.

**TABLE 4:** Wilcoxon signed rank test - Human-trust (HT) vs. Technology-trust (TT).

Comparison	Median	Std. Deviation	Sum of ranks	Asymp. Sig
RB-AHV-TT	6	.9222	575	
RB-AHV-HT	6	1.007	699	.515
RB-MHV-TT	5	1.071	623	
RB-MHV-HT	5	1.01012	602	.912
RB-AIV-TT	6	1.012	565	
RB-AIV-HT	6	1.181	660	.608
RB-TSV-TT	6	1.145	533	
RB-TSV-HT	6	1.3247	594	.736
SB-AHV-TT	6	.969	454	
SB-AHV-HT	6	.9685	365	.520
SB-MHV-TT	6	1.0288	983	
SB-MHV-HT	6	1.158	612	.098
SB-AIV-TT	6	.9507	658	
SB-AIV-HT	6	1.103	668	.960
SB-TSV-TT	5	1.1373	684	
SB-TSV-HT	5	1.167	443	.164

### 4.2 Body, voice and human-trust

A two-way ANOVA was performed to explore the main and interaction effect of agent body and voice on human-trust score. In the MHV-included dataset, there was no statistically significant difference in human-trust for agent body (F(1,788)=.536,p=.464) , as well as for agent voice (F(3,788)=1.617,p=.184) . There was also no significant interaction between agent body and voice (F(3,788)=1.317,p=.268) . The same was true for the MHV-excluded dataset for body (F(1,594)=.035,p=.852) , voice (F(2,594)=1.219,p=.296) , and interaction between body and voice (F(2,594)=.623,p=.536) . As a result, the null hypotheses H_2a, – there is no significant difference between human-trust scores for agent body, H_2b, – there is no significant difference between human-trust scores for agent voice, and H_2c, – the effect of agent body on human-trust score does not depend on agent voice, are accepted.

### 4.3 Body, voice and technology-trust

A Two-Way ANOVA was performed to explore the main and interaction effect of agent body and voice on technology-trust score. In the MHV-included dataset, there was no statistically significant difference in technology-trust for agent body (F(1,797)=.023,p=.881) . But there was a statistically significant difference in technology-trust for agent voice (F(3,797)=2.799,p=.039) . There was no statistically significant interaction between agent body and voice (F(3,797)=2.123,p=.068) . While the ANOVA showed a statistically significant difference for voice, neither Tukey HSD nor Games-Howell *post hoc* tests showed a statistically significant difference for any pairwise comparisons. The discrepancy was not examined further as in the MHV-excluded dataset, there was no statistically significant difference for either body (F(1,594)=1.503,p=.221) or voice (F(2,594)=2.982,p=.051) , or interaction effect between the two (F(2,594)=1.162,p=.314) . As a result, the null hypotheses H_3a–there is no significant difference between technology-trust scores for agent body, H_3b–there is no significant difference between technology-trust scores for agent voice, and H_3c–the effect of agent body on technology-trust score does not depend on agent voice, are accepted.

### 4.4 Body, voice and anthropomorphism

A Two-Way ANOVA was conducted to explore the main and interaction effect of agent body and voice on anthropomorphism score. For the MHV-included dataset, it was found that the residuals in six of the eight groups were not normally distributed. Anthropomorphism score differed statistically significantly for agent body (F(1,789)=11.585,p<.001) , and voice (F(3,789)=56.299,p=<.001) . There was no statistically significant interaction between agent body and voice (F(3,789)=.501,p=.682) . For the MHV-excluded dataset, residuals in four of the six groups were not normally distributed. Anthropomorphism score differed statistically significantly for agent body (F(1,594)=5.997,p=.015) , and voice (F(2,594)=43.548,p<.001) . There was no statistically significant interaction between agent body and voice (F(2,594)=.168,p=.845) . As a result, the null hypothesis H_4c–the effect of agent body on anthropomorphism score does not depend on agent voice, is accepted. The null hypotheses H_4a–there is no significant difference between anthropomorphism scores for agent body, and H_4b–there is no significant difference between anthropomorphism scores for agent voice, are rejected.

**FIGURE 2:**
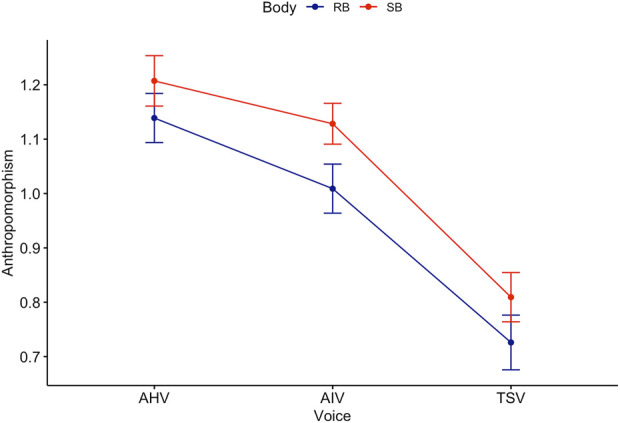
Trend lines for Anthropomorphism score across Body and Voice.

Games-Howell *post hoc* analysis on voice revealed a significant difference between all groups except TSV-MHV and AIV-AHV (see Figure 2). Mean level of anthropomorphism score increased from TSV to AIV (.30,95%−CI[.19,.42],p<.001) and from TSV to AHV (.41,95%−CI[.28,.53],p<.001) . It decreased from AIV to MHV (−.41,95%−CI[−.53,−.29],p<.001) , and increased from MHV to AHV (.51,95%−CI[−.39,.64],p<.001) . Games-Howell *post hoc* analysis on body revealed a significant difference between the robot (RB) and speaker (SB). Mean level of anthropomorphism increased from RB to SB (.10,95%−CI[.36,.18],p<.001) .

**FIGURE 3:**
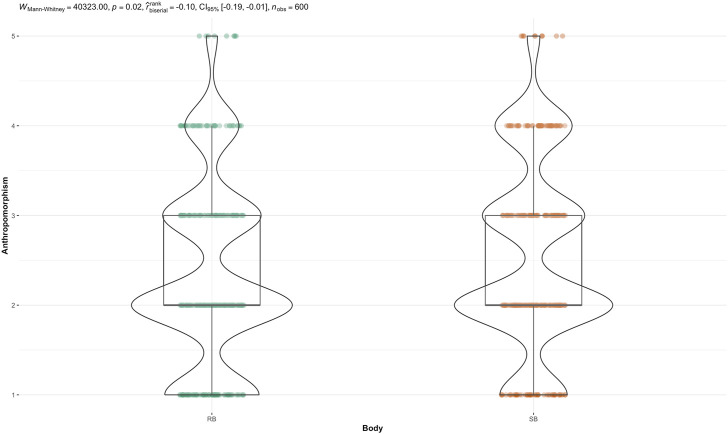
Mann-Whitney *U* test for Anthropomorphism score across Body.

**FIGURE 4:**
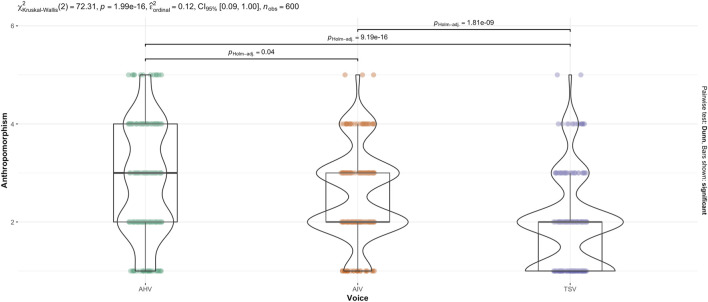
Kruskal–Wallis test for Anthropomorphism score across Voice.

The non-parametric, Mann-Whitney U test was performed to evaluate whether there was a statistically significant difference in the distribution of anthropomorphism perception across agent body (see Figure 3). The results indicated that speaker (SB) had statistically significant higher anthropomorphism rankings than robot (RB), (U=40323,p=.02) . A Kruskal–Wallis test was performed on voice (see Figure 4). It indicated that there was a statistically significant difference in anthropomorphism across voice, $\left(\chi^{2}\left(2,600\right)\right)=72.31,p<.001$ ). Holm-Bonferroni adjusted Dunn *post hoc* comparisons indicated that the TSV ranked significantly lower than AIV, (p<.001) , and TSV also ranked significantly lower than AHV, (p<.001) . There was also a significant difference between AIV and AHV, (p=.0.04) . This is mostly consistent with the two-way ANOVA result, except that the Games-Howell *post hoc* test did not find a significant difference between AIV and AHV.

### 4.5 Body, voice and animacy

A Two-Way ANOVA was conducted to explore the main and interaction effect of agent body and voice on animacy score. For the MHV-included dataset, it was found that the residuals in one of the eight groups were not normally distributed. There was a statistically significant difference in animacy for agent body, (F(1,789)=4.167,p=.042) , and voice (F(3,789)=32.866,p<.001) . There was no statistically significant interaction between agent body and voice, (F(3,789)=2.006,p=.112) . For the MHV-excluded dataset, residuals for all groups were normally distributed. There was no statistically significant difference in animacy for agent body, (F(1,594)=1.282,p=.258) , but there was a statistically significant difference for voice (F(2,594)=26.750,p<.001) . There was no statistically significant interaction between agent body and voice, (F(2,594)=2.189,p=.113) . As a result the null hypotheses, H_5a–there is no significant difference between animacy scores for agent body, and H_5c–the effect of agent body on animacy score does not depend on agent voice, are accepted. The null hypothesis H_5b–there is no significant difference between animacy scores for agent voice, is rejected.

**FIGURE 5:**
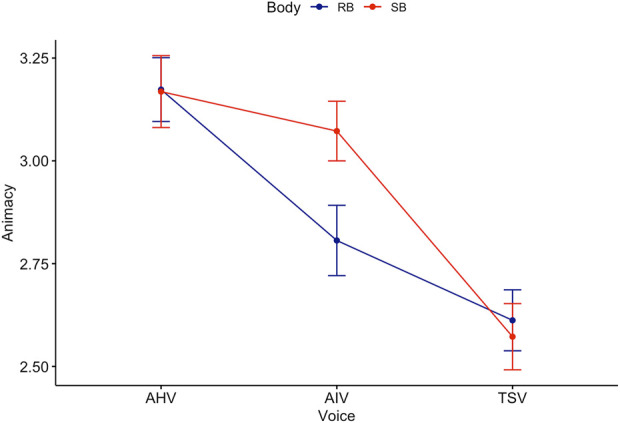
Trend lines for Animacy score across Body and Voice.

Games-Howell *post hoc* analysis on voice revealed a significant difference between all groups except TSV-MHV (see Figure 5). Mean level of animacy increased from TSV to AIV (.35,95%−CI[.14,.55],p<.001) , and from TSV to AHV (.58,95%−CI[.37,.78],p<.001) . It decreased from AIV to MHV (−.47,95%−CI[−.58,−.27],p<.001) , increased from AIV to AHV (.23,95%−CI[−.02,.44],p=.02) , and MHV to AHV (.70,95%−CI[−.49,.91],p<.001) .

**FIGURE 6:**
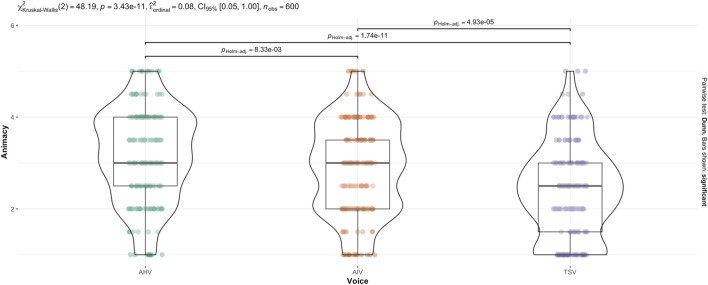
Kruskal–Wallis test for Animacy score across Voice.

The non-parametric, Kruskal–Wallis test indicated that there was a statistically significant difference in animacy across voice (see Figure 6), $\left(\chi^{2}\left(2,600\right)\right)=48.195,p<.001$ ). Holm-Bonferroni adjusted Dunn *post hoc* comparisons indicated that TSV ranked significantly lower than AIV, (p<.001) , TSV also ranked significantly lower than AHV, (p<.001) , and AIV ranked significantly lower than AHV, (p=.008) . This is consistent with the two-way ANOVA result.

### 4.6 Body, voice and likeability

A Two-Way ANOVA was conducted to explore the main and interaction effect of agent body and voice on likeability score. For MHV-included dataset, residuals in seven of the eight groups were not normally distributed. The level of likeability had no statistically significant difference by body, (F(1,789)=.042,p=.838) , but differed statistically significantly by voice, (F(3,789)=15.636,p<.001) . There was no statistically significant interaction between agent body and voice, (F(3,789)=.531,p=.661) . For MHV-excluded dataset, residuals in five of the six groups were not normally distributed. The level of likeability had no statistical difference by body, (F(1,594)=.362,p=.548) , but differed statistically significantly by voice, (F(2,594)=6.133,p=.002) . There was no statistically significant interaction between agent body and voice, (F(2,594)=.485,p=.616) . As a result the null hypotheses, H_6a–there is no significant difference between likeability scores for agent body, and H_6c–the effect of agent body on likeability score does not depend on agent voice, are accepted. The null hypothesis H_6b–there is no significant difference between likeability scores for agent voice, is rejected.

**FIGURE 7:**
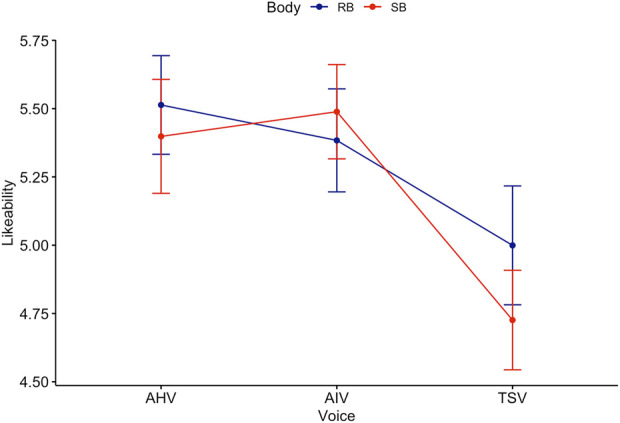
Trend lines for Likeability score acorss Body and Voice.

Games-Howell *post hoc* analysis by voice revealed a significant difference between all groups except AIV-AHV (see Figure 7). Mean level of likeability increased from TSV to AIV (.57,95%−CI[.08,1.06],p=.015) , decreased from TSV to MHV (−.57,95%−CI[−1.10,−.04],p=.030) , increased from TSV to AHV (.59,95%−CI[.08,1.10],p=.015) , decreased from AIV to MHV (−1.14,95%−CI[−1.65,−.64],p<.001) , and increased from MHV to AHV (1.16,95%−CI[−.64,1.69],p<.001) .

**FIGURE 8:**
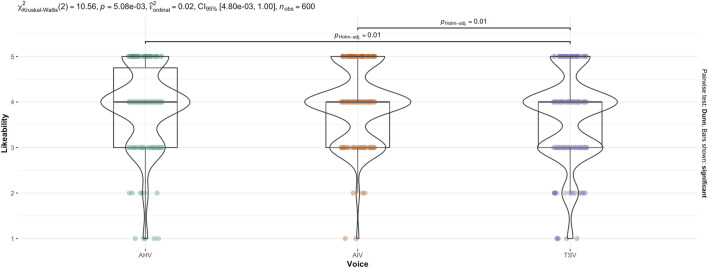
Kruskal–Wallis test for Likeability score across Voice.

The non-parametric, Kruskal–Wallis test indicated that there was a statistically significant difference in anthropomorphism across voice (see Figure 8), $\left(\chi^{2}\left(2,600\right)\right)=10.56,p=.005$ ). Holm-Bonferroni adjusted Dunn *post hoc* comparisons indicated that TSV ranked significantly lower than AIV, (p=.01) , and TSV also ranked significantly lower than AHV, (p=.01) . However, there was no significant difference between AIV and AHV, (p=1.0) . This is consistent with the two-way ANOVA result.

### 4.7 Body, voice and perceived intelligence

A Two-Way ANOVA was performed to explore the main and interaction effect of agent body and voice on perceived intelligence score. For MHV-included dataset, residuals in eight of the eight groups were not normally distributed. The level of perceived intelligence had no statistical difference by body, (F(1,789)=1.418,p=.234) , but differed statistically significantly by voice, (F(3,789)=2.806,p=.039) . There was no statistically significant interaction between agent body and voice, (F(3,789)=2.319,p=.074) . For MHV-excluded dataset, residuals in six of the six groups were not normally distributed. The level of perceived intelligence had no statistical difference by body, (F(1,594)=.157,p=.692) , or voice, (F(2,594)=1.192,p=.304) . There was no statistically significant interaction between agent body and voice either, (F(2,594)=2.687,p=.069) . As a result, the null hypotheses H_7a–there is no significant difference between perceived intelligence scores for agent body, H_7b–there is no significant difference between perceived intelligence scores for agent voice, and H_7c–the effect of agent body on perceived intelligence score does not depend on agent voice, are accepted.

Games-Howell *post hoc* analysis by voice revealed a statistically significant difference between the group AIV-MHV. Mean level of perceived intelligence decreased from AIV to MHV (−.78,95%−CI[−1.47,−.078],p=.022) .

### 4.8 Body, voice and perceived safety

A Two-Way ANOVA was performed to explore the main and interaction effect of agent body and voice on perceived safety score. In the MHV-included dataset, residuals in six of the eight groups were not normally distributed. The level of perceived safety had no statistical difference by body, (F(1,789)=.493,p=.483) , but differed statistically significantly by voice, (F(3,789)=12.524,p<.001) . There was no statistically significant interaction between agent body and voice, (F(3,789)=.816,p=.485) . In the MHV-excluded dataset, residuals in five of the six groups were not normally distributed. The level of perceived safety had no statistical difference by body, (F(1,594)=.029,p=.884) , but differed statistically significantly by voice, (F(2,594)=3.332,p=.036) . There was no statistically significant interaction between agent body and voice, (F(2,594)=.109,p=.897) . As a result the null hypotheses, H_8a–there is no significant difference between perceived safety scores for agent body, and H_8c–the effect of agent body on perceived safety score does not depend on agent voice, are accepted. The null hypothesis H_8b–there is no significant difference between perceived safety scores for agent voice, is rejected.

**FIGURE 9:**
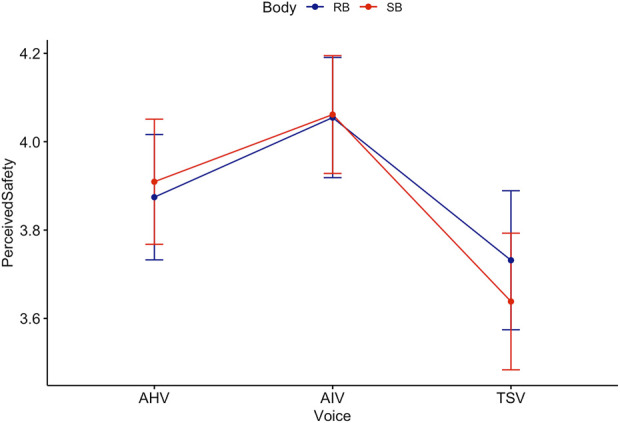
Trend lines for Perceived Safety score acorss Body and Voice.

Games-Howell *post hoc* analysis by voice revealed that mean level of perceived safety decreased from TSV to MHV (−.48,95%−CI[−.88,−.07],p=.013) , decreased from AIV to MHV (−.85,95%−CI[−1.23,−.47],p<.001) , increased from MHV to AHV (.68,95%−CI[.30,−1.07],p<.001) , and increased from TSV to AIV (.37,95%−CI[.03,.71],p=.029) (see Figure 9).

**FIGURE 10:**
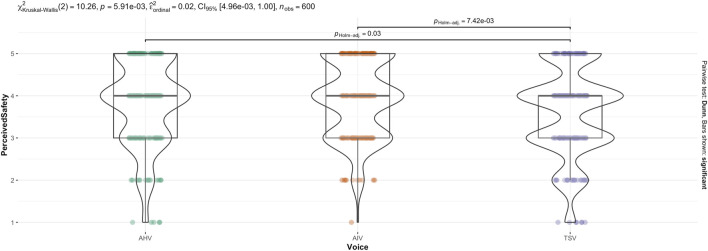
Kruskal–Wallis test for Perceived Safety score across Voice.

The non-parametric, Kruskal–Wallis test indicated that there was a statistically significant difference in anthropomorphism across voice (see Figure 10), $\left(\chi^{2}\left(2,600\right)\right)=10.26,p=.006$ ). Holm-Bonferroni adjusted Dunn *post hoc* comparisons indicated that TSV ranked significantly lower than AIV, (p=.007) , and also TSV ranked significantly lower than AHV, (p=.03) . However, there was no significant difference between AIV and AHV, (p=1.0) . This is mostly consistent with the two-way ANOVA result, except that the Games-Howell *post hoc* test did not find a significant difference between TSV and AHV.

## 5 Discussion

Overall, three primary findings are immediately apparent upon initial examination of the results, (1) lack of significant difference in human-trust and technology-trust, both within and between any of the conditions, (2) lack of significant difference in any of the independent variables (except anthropomorphism) across agent body (SB and RB), and (3) significant difference in a majority of the independent variables (outside of trust, and with the exception of perceived intelligence) across agent voice (AHV, AIV and TSV). It is also worth noting the lack of interaction effects between body and voice across any of the conditions. The results, for the most part, are consistent between the parametric and non-parametric tests, further indicating the robustness of the findings.

### 5.1 Trust

Given that both a smart speaker and a robot possess social cues by way of being able to speak and interact in human comprehensible ways, as well as perceived agency by way of being able to answer questions and perform tasks, the CASA paradigm would hold that both would elicit human social reactions (Reeves and Nass, 1996), and consequently trust–as conceived in HHI (Lankton et al., 2015). While the experiment did assume that both cases would produce both human-trust and technology-trust, it was not obvious whether differing levels of humanness significantly changed the type of trust placed in the social agent. If it did, that would contradict the CASA paradigm. From the results, we see that is not the case. Though the anthropomorphism results show that, indeed humanness of agent is perceived significantly differently across agent body and voice, those perceptive differences did not directly affect trust. All agents used in this study were anthropomorphic to varying degrees, while the results indicate that the degrees may not have had a significant effect on trust, generally high levels of trust (both human and technology) were recorded in all conditions as seen in Table 4. This is in-line with previous findings of a positive correlation between anthropomorphism and trust more broadly (Blut et al., 2021; Roesler et al., 2021).

The lack of differences in human-trust lends further weight to the CASA paradigm, especially considering that no differences were noted even in the MHV-included results. Despite the MHV audio being perceived as unclear, weird, and unpleasant, this did not significantly affect human-trust. This can be explained to some extent by the CASA paradigm. It would still be true that the agents in the MHV conditions possessed social cues by being able to communicate in spoken language, albeit poorly in comparison to other voices, and be able to perform tasks and display perceptive agency, meeting the two boundary conditions for CASA to apply (Gambino et al., 2020). And since the vignette depicts successful task completion by all the agents, it would explain the insignificance of human-trust differences across conditions. While previous studies have found that linguistic and behavioural traits had an effect on trust in voice-based agents (Hsu and Lee, 2023), seeing as these traits were maintained as constant with only a difference in the quality of humanness of voice, our results do not necessarily contradict these findings. Technology-trust on the other hand, performed slightly differently. While no significant differences were found in the MHV-excluded results, MHV-included results were significantly different, indicating that the MHV audio caused a loss of technology-trust, which is comprised of functionality, helpfulness and reliability. This shows that quality of voice-based interaction has a significant effect on technology-derived conception of trust. While technology-trust is stable despite significant differences in anthropomorphism perception, when voice-based interaction is not ideal, such as with an ineffective voice in the case of MHV, it negatively affected technology-trust. Although not part of the primary results of the study, this highlights that theoretical concepts underlying trust measurement need careful consideration as they highlight and obscure different aspects of trust.

In a previous study, Krantz et al. (2022) showed that when a robot had the ability to speak, the level of perceived trustworthiness the participants reported remained stable at a level similar to when the robot behaved perfectly, even when the robot failed. When the robot did not speak, the level of perceived trustworthiness reported by the participants dropped when the robot failed (Krantz et al., 2022). This showed that speech could be a mitigating factor in loss of trust in HRI. In our study, all agents possessed (simulated) the ability to speak, simply possessing this ability could have mitigated any potential loss of trust attributed to the characteristics (body and voice) of the agent, explaining the insignificance of differences in trust (of both types) reported across all the conditions. This hypothesis will need further examination. In addition, it could be that task-based agents are evaluated in terms of their ability to complete the task successfully, which might outweigh the influence of their characteristics on trust, and given that all agents perform the task in the exact same manner, it would explain the insignificant differences in trust. There is some evidence in other experiments to corroborate this argument (Hancock et al., 2011).

### 5.2 Anthropomorphism

Of all the dependent variables measured, anthropomorphism was the only one where both independent variables (body and voice) had a significant effect. This highlights that individuals are perceptively attuned to anthropomorphic features of an agent, and perceive anthropomorphism significantly differently both by changes in the physical presentation of the agent body, as well as the changes in the humanness of voice.

In terms of voice, TSV was perceived as less anthropomorphic than both AIV and AHV. This result was expected, as the text-to-speech (TTS) service used to create TSV audio is older, less advanced, and the result is noticeably artificial, which is by design. However, the differences in anthropomorphism perception between AIV and AHV are less clear. The parametric Games-Howell *post hoc* tests on the means of the transformed data, and the non-parametric Dunn *post hoc* tests on the medians of the non-transformed data, produce contradictory results, with the parametric tests finding no significant difference where the non-parametric tests found a significant difference. Given that the data fails to meet some assumptions for the parametric tests (despite robustness against non-normality, and the use of robust ANOVA), there may be reason to accept the non-parametric result in this instance, which would indicate that all three voices were perceived significantly differently in terms of anthropomorphism, with AHV being perceived as most anthropomorphic, followed closely by AIV, and TSV definitively being perceived as the least anthropomorphic. These results are largely in line with expectations based on literature (Lévêque et al., 2012; Kühne et al., 2020). In the MHV-included analysis, MHV was perceived as less anthropomorphic than both the AIV and AHV, with no significant difference from TSV. This shows that less-than-ideal voice quality negatively affects the perception of anthropomorphism in agents.

In terms of body, the speaker was perceived as more anthropomorphic than the robot. This is confirmed by both the parametric and non-parametric tests. At first glance, the result is unexpected. Smart speakers (especially ones without a screen to present a virtual body, such as in our study) are generally considered disembodied agents in HAI (Bonfert et al., 2021; Lee et al., 2006), and research suggests that embodiment and anthropomorphism are positively correlated (Roesler et al., 2023; Kiesler et al., 2008). As a result, it would be reasonable to assume that, all other things being equal, a humanoid robot would be perceived as more anthropomorphic than a speaker when using the same voice to communicate. However, our results present the contrary. One explanation could be that there was a perceived mismatch of expectations between the robot’s appearance and its performance. Research has found that humans form certain expectations regarding social robot’s overall abilities by displaying a few human-like qualities, such as speech, and when the expectations are mismatched with performance, it can lead to negative overall effects (Kwon et al., 2016; Paepcke and Takayama, 2010). In our experiment, the humanness of the body of the robot may have created greater expectations of human-like capabilities, resulting in less anthropomorphism perception when the robot fails to meet this expectation. This expectation-gap is of less concern for the speaker, as the speaker does what it would likely be expected to do. Another explanation could be that design of the robot had an impact on perception. In chatbots, it was found that highly intelligent disembodied agents were perceived as more human-like than highly intelligent (virtually) embodied agents with poorly designed appearances (Kim and Im, 2023). Seeing as this study was video-based, it may be reasonable to interpret the results through these findings on virtual embodiment. However, our results are contradictory to some studies that find that robots with voices were perceived as more anthropomorphic than voice-only agents Luo et al. (2023). This may be a reflection of the differences between video-based and direct interaction methods.

The lack of significance in interaction effects between body and voice indicates that body anthropomorphism and voice anthropomorphism are distinct phenomena, and that they individually influence the perception of the agent. This lends some empirical support to the multidimensional nature of anthropomorphism perception. Measuring anthropomorphism perception as a singular phenomenon risks missing these underlying dimensions and their individual effects on perception of an agent.

### 5.3 Social characteristics

We categorise the rest of the Godspeed scales used (as dependent variables) – animacy, likeability, perceived intelligence and perceived safety, as pertaining to ‘social characteristics’ for ease of discussion. Agent voice had a significant effect across these dependent variables while agent body had no significant effect (with the exception of perceived intelligence where neither had an effect). AHV was perceived as the most animate, and TSV as the least. TSV was perceived as the least likeable, but there was no significance between likeability between AIV and AHV. TSV was also perceived as the least safe. Previous studies have indicated that anthropomorphism may not directly impact trust, rather, increase social attraction, which in turn reinforces trust (Chen and Park, 2021). Anthropomorphism of voice in this study does result in greater perception of social characteristics which could be considered an aspect of social attraction, however, that does not impact trust. In the MHV-included results, voice also had a significant effect on perceived intelligence, showing that quality of voice interaction impacts perception of intelligence of an agent, and that it may be of relevance to disembodied agents as well, arguably extending the findings from previous studies pertaining embodied agents (Schneiders et al., 2021).

Previous research employing the same Godspeed scales has found that a speaking robot was seen as more lifelike, likeable, and as having higher perceived intelligence than a non-speaking robot, regardless of whether it displayed faulty behaviour (Krantz et al., 2022). The results from our study further extend these findings and show that not only does presence or absence of voice have an impact on anthropomorphism, likeability, and perceived safety, but the qualitative nature of voice, has a significant impact as well, with perceptively more human voices performing better in terms of perception of social characteristics. This result is of particular relevance for agent design, as it implies that voice design may have significantly more impact on user perception than the design of the physical agent.

### 5.4 Contribution

Overall, the study makes three contributions to the state of the art in HAI. The results show that (1) body and voice both contribute to anthropomorphism perception independently from one another, serving as empirical evidence for the multidimensionality of anthropomorphism, (2) voice has a stronger impact on the perception of social characteristics of an agent compared to body, and (3) provided successful interaction, users perceive both human and technology conceptions of trust in agents with no significant difference and irrespective of the physical or voice attributes. However, these results should be considered in light of the context and limitations of the study.

### 5.5 Limitations

Interaction complexity is a significant limitation of most studies in HAI. Human-agent interaction is highly complex, and can vary significantly based on context, making it difficult to generalise findings across different settings. In this study, through a vignette, we depict a ‘travel agent’ context in order to convey a relatively low-stakes setting for an interaction, but that means that the results may not reflect high-stakes settings, like banking, for example. The choice of agents and voices used also affects perception and interaction. For example, one study found, using Furhat (Al Moubayed et al., 2012) in a workplace context, a significant difference in user perception of trust between robot and smart speaker (Robb et al., 2023), which was not the case in this experiment using Epi in a service context.

The video-based, pseudo-interaction research design is a limitation in terms of Ecological validity. Since the participants do not directly interact with the agents, rather they watch videos of simulated human-agent interaction, the results from such a study may not reflect live-interaction (Bainbridge et al., 2011). Although, arguments to the contrary have also been made (Woods et al., 2006; Honig and Oron-Gilad, 2020). It can also be argued that the results may be more directly applicable to virtual agents than physical agents, however, the insights may still be relevant. This is an ongoing debate in HAI methodology.

Measurement validity might be considered a limitation. While the scales chosen are widely-used, many of the subjective components such as trust and anthropomorphism do not as-yet have ideal quantitative measures, as the theory which they are founded on is still debated. For example, it is argued whether the Competence/Ability, Benevolence and Integrity conceptualisation of human-trust encapsulates the experience of trust (Baer and Colquitt, 2018).

### 5.6 Future research

Several variations of the study could be performed to provide further insights into the effect of body and voice anthropomorphism on perception of the agent in human-agent interaction. Firstly, changing the body of the agents used could highlight the impact of variations in embodiment. Altering the voices used (for example, quality of humanness, gender, accent or speed) could lead to a more nuanced understanding of the impact of voice in perception of an agent. And using different scales of measurement derived from different theoretical conceptualisations of trust, anthropomorphism, and social characteristics, could further highlight different aspects of the phenomenon being measured. The study could also be reproduced employing qualitative and/or live-interaction methods to gain further insights into the subjective experience of human-agent interaction, and to contrast the qualitative results with the quantitative ones presented in this study.

The results from this study lend some empirical weight to a nuanced understanding of anthropomorphism by highlighting two of the dimensions, perceived simultaneously and independently, through body and voice. The results also highlight the significant role played by voice in agent perception, but further empirical research into the relationship between voice, embodiment, and anthropomorphism is needed to draw generalisable conclusions about the role of voice. At the same time, there are several other agent modalities of communication that could also possess anthropomorphic qualities and influence perception, such as gestures and tactile interaction. Further research in this direction is needed to expand our understanding of anthropomorphism perception in humans, its dimensions, and relationship with various agent modalities. Additionally, developing methods of measurement for these dimensions of anthropomorphism is also needed.

## 6 Conclusion

In this paper, we presented an online, video-based, human-agent interaction experiment, conduced using a speaker and robot that each employed four different levels of humanness of voice, to study the (1) type of trust (human-trust vs. technology-trust) exhibited by humans in social agents, (2) understand whether modalities of agent (body and voice) have an impact on the type of trust exhibited by humans, and (3) study the impact body and voice modalities have on anthropomorphism perception–to make inferences about the dimensions of anthropomorphism. We found that the participants exhibited both human-trust and technology-trust in agents, with no significant difference between the two, within and between all conditions. Body and voice had no significant impact on either type of trust, despite both (independently) having a significant impact on anthropomorphism perception. Voice was a significant factor in perception of social characteristics outside of trust and perceived intelligence, while body was not.

The results broadly indicate that trust is a relatively stable construct, despite varying anthropomorphism, provided successful agent interaction. Body and voice both independently lead to anthropomorphism perception, highlighting different dimensions of anthropomorphism–arising from different modalities of agent. And voice is a significantly stronger factor than body in influencing the perception of the agent. These results have implications for agent design, where voice stands out as an important modality to consider. Furthermore, the results expand upon theory on anthropomorphism by providing weight to the argument for a multidimensional understanding of anthropomorphism.

## Data Availability

The raw data supporting the conclusions of this article will be made available by the authors, without undue reservation.

## Footnotes

1 HAI and HRI are adjacent fields that both deal with human interaction with agents. While HAI pertains to agents more broadly, including but not limited to robots, chatbots and voice assistants, HRI more narrowly focuses on robots alone. In this paper, we use the term agents (even when referring to robots) and use the broader term HAI (interchangeably with HRI) as our study pertains to trust and anthropomorphism in both voice assistants and robots.

2 We refer to multidimensionality of anthropomorphism in the perceptive experience of humans, and multimodality of agents in their design/characteristics to interact using various modes of communication such as gestures, speech, etc. As a result, we surmise that multimodality of agents is correlated to multidimensionality of anthropomorphism in perception.

3 Voice, speech, and language are distinct yet related concepts. In this paper, given the context of vocal agents and their ability to speak using human language, we refer to the combined attributes of these concepts when using the words voice or speech.

4 ‘TSV’ and ‘AIV’ voices are both generated using text-to-speech (TTS) algorithms, which is a technical term. The names for the conditions ‘text-to-speech’ and ‘AI generated’ are not used in a technical sense, they are mainly intended to make a clear distinction between the conditions, and are derived from how the services used in creating the voices are marketed (see Section 3.3.1).

5 The modified human voice ‘MHV’ (and the associated conditions RB-MHV and SB-MHV) were found to be flawed during analysis (see Section 3.3.2). Data analysis was performed both including and excluding the variable and both are reported.

6 The hypotheses remain applicable both including and excluding MHV.

7 A subscription to the public-use product was purchased to generate the audio files.

8 As discovered during analysis (see Section 3.5.2) MHV was a flawed condition, as the edited voice was perceived to be unpleasant, explaining the low rank here. We address the design flaw in Section 3.5.2.

9 Non-parametric alternatives to one-way ANOVA for the two dependent variables body and voice are deemed sufficient where no interaction effects are found in the two-way ANOVA results.
