# Supplementary material for: Talking body: the effect of body and voice anthropomorphism on perception of social agents
Source: Front Robot AI. 2024 Oct 9;11:1456613. doi: 10.3389/frobt.2024.1456613 (PMC11496039; doi:10.3389/frobt.2024.1456613)
Supplement: Supplementary file 1 [file DataSheet1.pdf]

Supplementary Material

1 APPENDIX 1: VIGNETTE

Participants were provided the following information for context before watching the videos. The audio for the videos were created using the script below to depict a human-agent interaction.

1.1 Context

The video is of an interaction between Otto (a robot/smart speaker assistant – *changed as applicable for the condition*) and a user (a woman). The user is planning a weekend getaway to Aarhus, Denmark, using the help of Otto (a smart, travel assistant for the city of Aarhus) to make bookings for her trip. Listen to the interaction. Pay attention as you will have to answer questions about the interaction later.

1.2 Conversation

User: Hey, I need help with something

Agent: Sure, what can I do for you?

User: I want to go on a weekend trip to Aarhus and need to book accommodation and organise the sites I want to visit

Agent: Okay, let me get some details first. When are you planning to visit Aarhus?

User: On 21st and 22nd of October

Agent: Okay. What is your budget per night for accommodation?

User: Around 1500 danish crowns per night

Agent: Alright. Do you know where you want to stay?

User: I was curious about The Daneland but I'm not sure if I can afford it?

Agent: Okay, let me check. I see that The Daneland has a single room for 1290 danish crowns per night. Does that work for you?

User: Yes, it does.

Agent: Great, I will make a reservation in your name.

User: I also want to visit the Aros museum on the 21st, can you buy me a ticket for that?

Agent: Okay, I will make a booking in your name for the Aros museum on the 21st. Is there anything else I can do for you?

User: Yes, I need a weekend public transport pass to get around.

Agent: Alright, the 48 hour public transport pass in Aarhus costs 449 danish crowns. Do you want to purchase this?

User: Yes.

Agent: Done. Is there anything else you need?

User: Yea, I was curious about the public library in Aarhus. Is it open on the weekend? Can I visit on the 22nd?

Agent: The public library called Dokk1 is part of the renovated harbour front in the city. On weekends it is open from 10:00 to 16:00

User: Great, thanks. Is there anything else I should do while in Aarhus?

Agent: Aarhus has many great attractions. You could visit the Moesgaard museum located just outside the city or walk around the Latin Quarter. The botanical gardens are beautiful in Autumn. The city also has many cosy cafes and restaurants. Do you want specific suggestions?

User: Can you suggest one cafe and one restaurant I must try?

Agent: The Salling roof top cafe lets you enjoy an amazing panoramic view of the city. And Aarhus Street Food has a wide selection of cuisines from all over the world to try. Do you need help with anything else?

User: No, that'll be it I think.

Agent: Great, I will direct you to the checkout on your connected smartphone.

User: Alright, thanks!

Agent: You're welcome. Let me know if you need anything else.

2 APPENDIX 2: ADAPTED TRUST SCALES AND ITEMS

The human-trust is comprised of three sub-scales – integrity, competence, and benevolence, and the technology-trust is comprised of three sub-scales – functionality, helpfulness, reliability.

2.1 Human-trust

2.1.1 Integrity

Rate the following statements on a 7-point scale from strongly disagree (1) to strongly agree (7).

- Otto is truthful in its dealings with the user
- Otto is honest
- Otto keeps its commitments

2.1.2 Competence

Rate the following statements on a 7-point scale from strongly disagree (1) to strongly agree (7).

- Otto is competent and effective in vacation planning
- Otto performs its role of vacation planning very well
- Otto is capable and proficient at vacation planning

2.1.3 Benevolence

Rate the following statements on a 7-point scale from strongly disagree (1) to strongly agree (7).

- Otto would act in the user's best interest
- Otto would do its best to help the user if they need help

- Otto would be interested in the user's well-being, not just its own

2.2 Technology-trust

2.2.1 Functionality

Rate the following statements on a 7-point scale from strongly disagree (1) to strongly agree (7).

- Otto has the functionality the user needs for vacation planning
- Otto has the features the user requires for vacation planning
- Otto has the ability to do what the user wants it to do when vacation planning

2.2.2 Helpfulness

Rate the following statements on a 7-point scale from strongly disagree (1) to strongly agree (7).

- Otto supplies the user's need for help through a help function
- Otto provides competent guidance (as needed) through a help function
- Otto provides whatever help the user needs

2.2.3 Reliability

Rate the following statements on a 7-point scale from strongly disagree (1) to strongly agree (7).

- Otto is very reliable
- Otto does not fail the user
- Otto is extremely dependable
